# IPM Decisions Platform – a Pan-European online platform hosting decision support systems and associated resources for integrated pest management.

**DOI:** 10.12688/openreseurope.21411.1

**Published:** 2025-10-16

**Authors:** Mark Ramsden, Berit Nordskog, Tor-Einar Skog, Dave Skirvin, Angelo Marguglio, Antonio Caruso, Christophe Pradal, Lise Jorgensen, Mette Sonderskov, Nikos Georgantzis, Marko Debeljak, Jurij Marinko, Harm Brinks, Bjorn Andersson, Ilias Travlos, Eleanor Dearlove, Neil Paveley

**Affiliations:** 1 ADAS, Cambridge, UK; 2 NIBIO Division of Biotechnology and Plant Health, As, Akershus, Norway; 3 Engineering ingegneria informatica S.p.A., Palermo, Italy; 4 CIRAD Departement Systemes biologiques, Montpellier, Occitanie, France; 5 Aarhus University, Aarhus, Central Denmark Region, Denmark; 6 Burgundy School of Business, Dijon, France; 7 Jožef Stefan Institute, Ljubljana, Slovenia; 8 Delphy B.V., Wageningen, The Netherlands; 9 SLU, Swedish University of Agricultural Sciences, Uppsala, Sweden; 10 Agricultural University of Athens, Athens, Attica, Greece; 11 AHDB, Stoneleigh, UK

**Keywords:** Integrated Pest Management, Decision Support Systems, Decision Tools, Digital Infrastructure, Sustainable Pesticide Use

## Abstract

Crop protection and pest management are major economic and environmental concerns throughout Europe. The consultation of decision support systems (DSS) to guide decisions relating to Integrated Pest Management (IPM) is one of the key principles of IPM, reducing the ambiguity around potential risks to crop health. ‘Pests’ in this context include invertebrate pests, weeds and pathogens. The impact of DSS can be limited by a lack of awareness of DSS availability, inconsistencies in the user functions of different DSS, regional fragmentation of access, and a lack of transparency of the origin, validity, and benefits of DSS. Failure to address these limitations undermines trust in IPM DSS and leads to a reluctance of farmers and advisors to invest time in consulting multiple DSS sources as part of their agronomic decision toolbox. The EU-funded IPM Decisions project (Grant agreement ID: 817617) addressed these limitations by creating a Europe-wide free-access online platform. The IPM Decisions platform was designed in consultation with farmers, advisors and wider stakeholders to increase access to and uptake of IPM DSS integrated within it. It offers an end-point for IPM researchers and DSS developers to make adapted and novel DSS available to users, and provides a ‘one-stop shop' for farmers and advisors looking to consult free access or paid IPM DSS. Dedicated dashboards within the platform facilitate farm set up, consultation of DSS, comparison of DSS outputs, and adjustment of model parameters for adaption to different pests/regions. The IPM Decisions digital infrastructure enables easy integration of models and data with external platforms, providing a framework for accessing and sharing models and data between researchers and developers. The platform therefore provides both a ready to go user interface for new DSS, as well as the infrastructure to support and connect existing and future user interfaces.

## Plain language summary

The IPM DECISIONS project has developed an online platform that makes a variety of Decision Support Systems (DSS) accessible, which provide pest risk forecasts for a range of invertebrate pests, weeds and dieases in Europe. The platform automatically selects the most appropriate open-access weather data for the users farm location, and each DSS provides summary guidance on appropriate next steps.

The platform was designed based on close interactions with farmers and advisors across Europe, and forecast systems can be added or adapted by researchers and developers across Europe. Standardised formats defined in the platform ensure that models and outputs are provided in a consistent way. Within the platform, this supports users in selecting and consulting DSS in minutes.

IPM DSS have been shown to improve targeting of pest infestation management, and avoid application of unnecassary pesticide treatements. By consulting DSS, farmers and advisors are able to reduce the uncertainty around potential crop infestations, and so apply treatements according to need with greater confidence.

DSS are available through the platform in a number of ways. Some are fully intergrated, some are adapted from more detailed models in other platforms. In each case, details of where the model was developed, and any assumptions or limitations of use are detailed.

As part of developing the platform, several potential barriers and incentives to DSS consultation have been identified, along with pathways to improving access to and update of IPM DSS in Europe. Recomendations include supporting research and devlopment of novel IPM DSS, improving digital infrastructure and training within the agricultural sector, and supporting wider demonstraiotns of IPM DSS in practice.

The IPM Decisions platform has established a low-maintenance, high impact platform and supporting digital infrastructure to advance consultation of IPM DSS in Europe.

## Decision Support Systems for Integrated Pest Management

Societal demand to reduce the risks associated with the use of plant protection products (PPPs, hereafter referred to as pesticides) remains strong. Practical solutions are required to deliver this, whilst protecting the productivity and competitiveness of European agriculture. Integrated pest management decision support systems (IPM DSS) enables holistic approaches to reduce the need for pesticides and guides treatments that are appropriate to the reduced need. Reducing pesticide use without adverse impacts on productivity and competitiveness requires two steps:

1.   Reducing the need for pesticides by integrating non-chemical control measures

2.   Treating crops with pesticides according to the reduced need.

The IPM Decisions (2019) project addressed the second requirement by creating an online platform hosting decision support systems (DSS) for integrated pest management (IPM), supporting improved targeting of pesticide treatment according to need. Increasing the use of DSS throughout Europe will help to fulfil the requirements in Article 14 of Directive 2009/128/EC of the European Parliament and the Council on the Sustainable Use of Pesticides Directive (SUD). A sister project IPMWORKS (2020) established an IPM demonstration farm network, to showcase the benefits of IPM and the application of integrated non-chemical control measures to reduce the need for pesticides. IPM DSS reduce the ambiguity around the risk posed by pests to crops, supporting farmers and advisors to avoid unnecessary interventions (principles 2 and 3) and effectively target interventions where crops are at risk (principles 6 and 8). DSS that guide the use of non-chemical control measures or aid choice of chemical control, address principles 1, 4, 5 and 7. Synergies between these principles have been established, demonstrating, for example, that targeting treatments according to need (principle 6) is a key anti-resistance strategy (principle 7), and that non-chemical IPM approaches, such as the use of disease resistant crop varieties (principle 1), increases the economic benefits to farmers from the use of DSS. There are a diverse range of DSS including pest monitoring, economic thresholds, forecasts of pest prevalence and damage, and systems for comparing treatment options, all contributing to the key principles of IPM as summarized in the SUD. IPM DSS are an important tool in supporting pesticides use ‘according to need’, and accounting for the reduced need for treatment from integrating genetic, cultural and chemical control. This has benefits in both reducing the negative impacts of over-reliance on synthetic chemical pesticides and increasing the environmental and economic sustainability of food production systems ( Jørgensen *et al*., 2017; te Beest *et al.*, 2009). The use of DSS is a key component in enabling transition towards low pesticide use, in line with the European Commission’s Farm to Fork strategy’s target of a 50% reduction by 2030 in the use and risk of chemical pesticides and the use of more hazardous pesticides.

There are some excellent examples of IPM DSS, which are well tested and implemented ( Jørgensen, 2020), but the impact of DSS has been constrained by:

- Regional fragmentation of DSS development and user communities, limiting awareness and access to what is available
- Lack of quantification of the economic and environmental benefits of using DSS - often limited by access to sufficiently large datasets across a wide range of sites and seasons
- Inadequate testing of DSS for accuracy and predictive value (also data limited)
- Short-term funding curtailing long-term updating and user support
- DSS addressing single pests, whilst farmer decisions need to account for multiple pests
- DSS which are insufficiently risk-averse to meet farmer needs for protection against occasional severe epidemics causing large economic losses

Developers of DSS put a large investment into producing systems that are provided to end users free or as a paid service. Developers undertake common activities, such as optimising DSS, testing performance, maximising uptake by users and ensuring reliable decisions in extreme seasons (to ensure continued use). Some DSS developers have already decided that these activities are better achieved by joining together into wider geographic collaborations and platforms. The IPM Decisions platform facilitates this collaborative approach, by enabling sharing of resources between regional developers. The platform provides tools for comparing and adapting DSS from alternate sources, and for the creation of new DSS based on existing and novel approaches.

The aim of this open letter is to introduce the IPM Decisions platform, and how it supports farmers, advisors, researchers, DSS developers and policy makers in efforts to transition towards reductions in pesticide applications through advanced holistic IPM strategies.

### Development of the IPM Decisions platform

Development of the platform began in 2019 and the first open version was launched in September 2022. The platform is free to use and connects users with DSS developed across Europe on arable, vegetable, soft fruit, top fruit, and grapevine crops. It is the first open-access pan-European ‘click and go’ marketplace for IPM DSS, and integrates the meteorological data to run them ( https://www.platform.ipmdecisions.net/ IPM Decisions Platform, 2025). The platform was not designed for, or tailored to any particular IPM DSS, rather it hosts DSS from external providers. The sections below describe integration of these external DSS onto the platform and provision of platform resource to external sites. Annual planned updates to the backend and user interface ensure maintenance of the state-of-the-art design and function. Additional DSS can be integrated at any point by following the process outlined below.

***Platform design and development***

An incremental and iterative approach to development was used, starting from definition of requirements to DSS design (through the use of mock-ups), to technical specification definitions, up to their technical implementation (development of end-user web interfaces). The iterative process was facilitated by three rounds of multi-actor workshops across 12 participating countries, to which institutions, companies involved in farm advisory services, and farmers and advisors were invited. Initial workshops in January and February 2020 were face-to-face, subsequent workshops were held online in December 2020 – February 2021 and April-May 2022. All workshops were conducted in the national language. During the workshops, attendees were given a short introduction to the aims of the platform, and a demonstration of the current approach and proposed interface. Feedback was collected through a series of surveys, collecting both descriptive data about the responder and feedback on the platform design. The approach ensured the final version of the platform reflected the true needs of farmers, advisors, researchers and DSS developers, and enabled assessment of the incentives and barriers to the uptake of IPM DSS ( Marinko *et al.*, 2025a).

**Figure 1.**
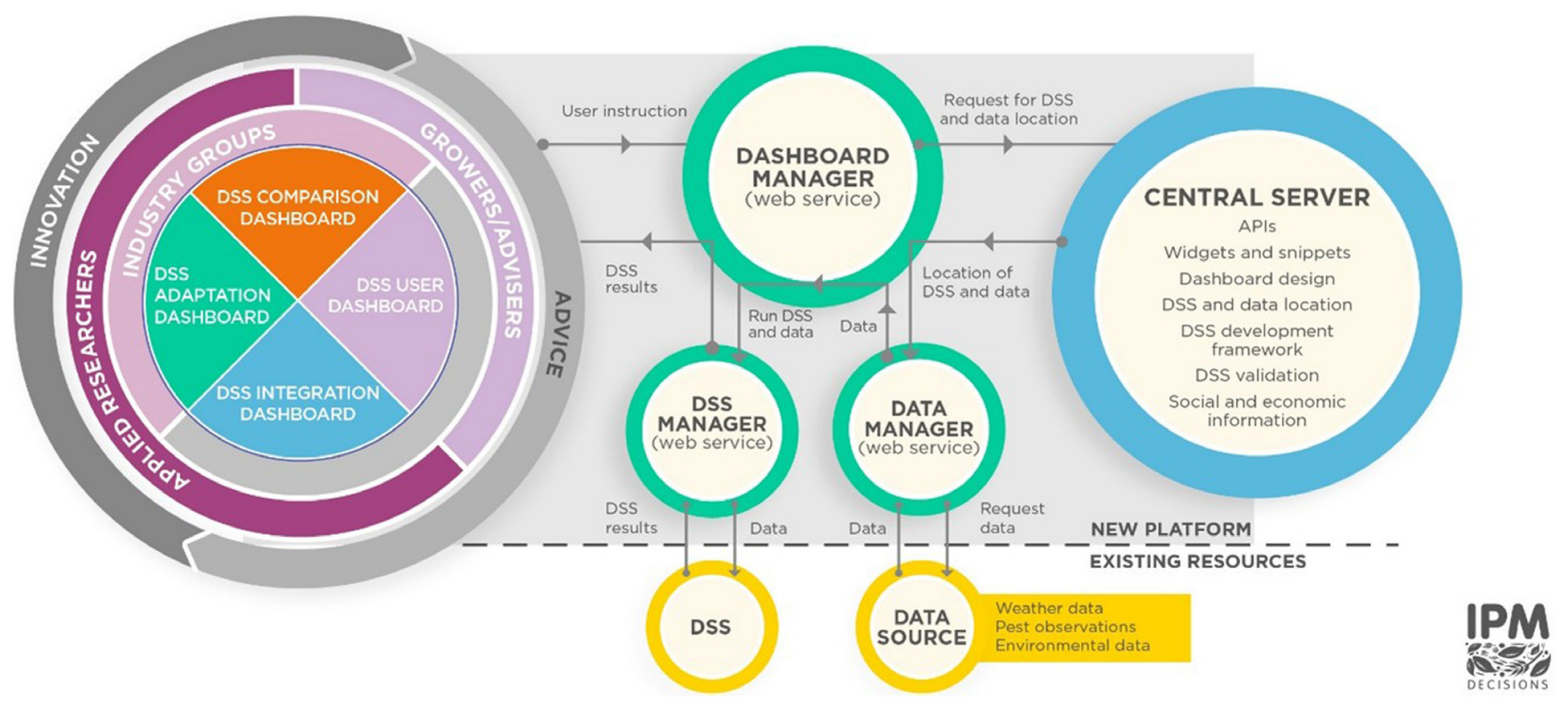
Overview of components of the IPM Decisions Platform.

The flow diagram ( Figure 1) illustrates the components of the Platform and their connections. A central server acts as a repository containing DSS information, data information, application programming interfaces (APIs), and source code. This repository includes the web services used to manage the Dashboards and their information requirements, widgets and snippets (small pieces of computer code that provide core functionality such as visualisation via maps, graphs and tables, or forms for user input) used in the Dashboards, Dashboard web page designs and tools for DSS evaluation, adaptation and development. A repository of central source code is available on GitHub ( IPM Decisions, 2022). This repository provides anyone with the ability to retrieve the latest version of the code and see all the development history for the code. The platform is currently available in 12 languages: Danish, Dutch, English, Finnish, French, German, Greek, Italian, Lithuanian, Norwegian, Slovenian, and Swedish. This includes both the content of the user interface, and the descriptive metadata associated with each DSS hosted on the platform.

During the IPM Decision project (June 2019 – May 2024), support has been available for the development of web and data formatting APIs, and for translation of associated metadata and documentation. After the end of the project, DSS developers need to fund these elements in order to integrate their DSS into the platform. The level of funding required depends on the number and complexity of DSS, and integration type, and is discussed on a case by case basis with platform developers.

### Providing access to weather data

Many DSS require access to weather data and additional metadata (such as crop information) to calculate pest risk. During the development of the IPM Decisions platform, suitable sources of weather data and/or metadata were identified and software created to enable the DSS hosted on the platform to access them. The data must meet the input requirements of a wide range of models, be locally relevant, quality controlled and easy to find for the user. These requirements were met by the establishment of two Application Programming Interfaces (APIs), the weather service and the DSS service ( Huitu *et al.*, 2025). The Weather API is a web service providing a catalogue of weather data sources and a standard for weather data exchange ( Skog *et al.*, 2024), while the DSS API is made for interaction with a repository of model metadata. Using Open-Meteo.com’s APIs (2025) enables the service to provide hourly weather data of agrometeorological relevance with a 1–7km resolution covering Europe. This provides a more reliable pathway to getting coverage than exclusively integrating local weather data sources. Additional local sources of climate data can be further integrated, providing increased detail for each region. Some weather parameters needed for DSS (such a leaf wetness duration; LWD) are rarely provided through public weather services. A selection of LWD models were tested on weather data representing different climatic zones of Europe. Two models were verified and included in the Weather API: One that simply uses RH > 88% as the threshold, and one that is based on machine learning (LSTM) that uses RH, rainfall, temperature and wind speed.

The quality of weather data sources for different countries/regions of Europe varies greatly. For some regions, data is provided from high quality weather stations or a high-resolution gridded dataset, while in other regions access may be limited to course gridded data of stored weather forecasts. For each country or region, the available data sources have been rated. When the platform requests weather data for a user's farm from the weather API, the API uses its catalogue of various weather data sources to select data source(s) providing the best data for the given location. For a given location, the API attempts to get the requested data, e.g. hourly rainfall, temperature and relative humidity for a given period during a growth season, from the highest rated data source. If all the requested data can be delivered from that source, then the API returns them to the platform. If for instance only rainfall and temperature can be collected from the highest rated data source, the API attempts to collect relative humidity data from the lower rated data sources and collates the data and returns them to the platform. If a special weather parameter like for instance leaf wetness is requested, then it might happen that no weather data sources near the farm can provide it. In that case, the API resorts to calculating the parameter from the parameters that are available in the dataset. Any warnings associated with missing weather data are provided to the user within the DSS Use dashboard.

### Process for integration of DSS onto the platform

The development of novel IPM DSS usually focuses on understanding and describing pest/crop ecology, to develop predictive models. Empiric and mechanistic models are the most relevant models in supporting decision-making in IPM ( Caffi *et al.*, 2007). Empiric models, also called data-based models, organize data, and standardise their relationship in terms of mathematical or statistical representations (e.g., correlation between pest abundance and air temperature). These provide useful insight to explore the relationships within a system that are unknown or poorly known. Mechanistic, or process-based, or fundamental, models describe a process (e.g., pest population dynamics/epidemics) based on the underlying functional mechanisms of the process. Model development is often the result of research focussed funding streams and has not included development of appropriate user interfaces. The IPM Decisions platform enables researchers to make their work available to users, without the need for bespoke Graphical User Interface (GUI) development. The process for integration of new DSS onto the platform has been made intentionally simple, lowering the ‘entry threshold’ for new DSS developers to integrate their DSS and reach users.

To include a DSS on the platform, contact is first established and nominated contacts are established between the IPM Decisions platform developers and the DSS developers, and the suitability of the DSS is reviewed. DSS vary widely in their characteristics, so there are four options for how a DSS can interact with the Platform:

1. DSS delivered to user via Dashboard (‘ *Full integration*’)
2. Simple pest risk summary from DSS delivered to user via Dashboard. Users passed to existing DSS interface for full DSS functions (‘ *Partial integration*’)
3. DSS utilises resources from the Platform and delivers DSS to users through its own interface (‘ *Utilise resources*’)
4. Users access DSS via a link on the Dashboard (‘ *External Link*’)

**Figure 2.**
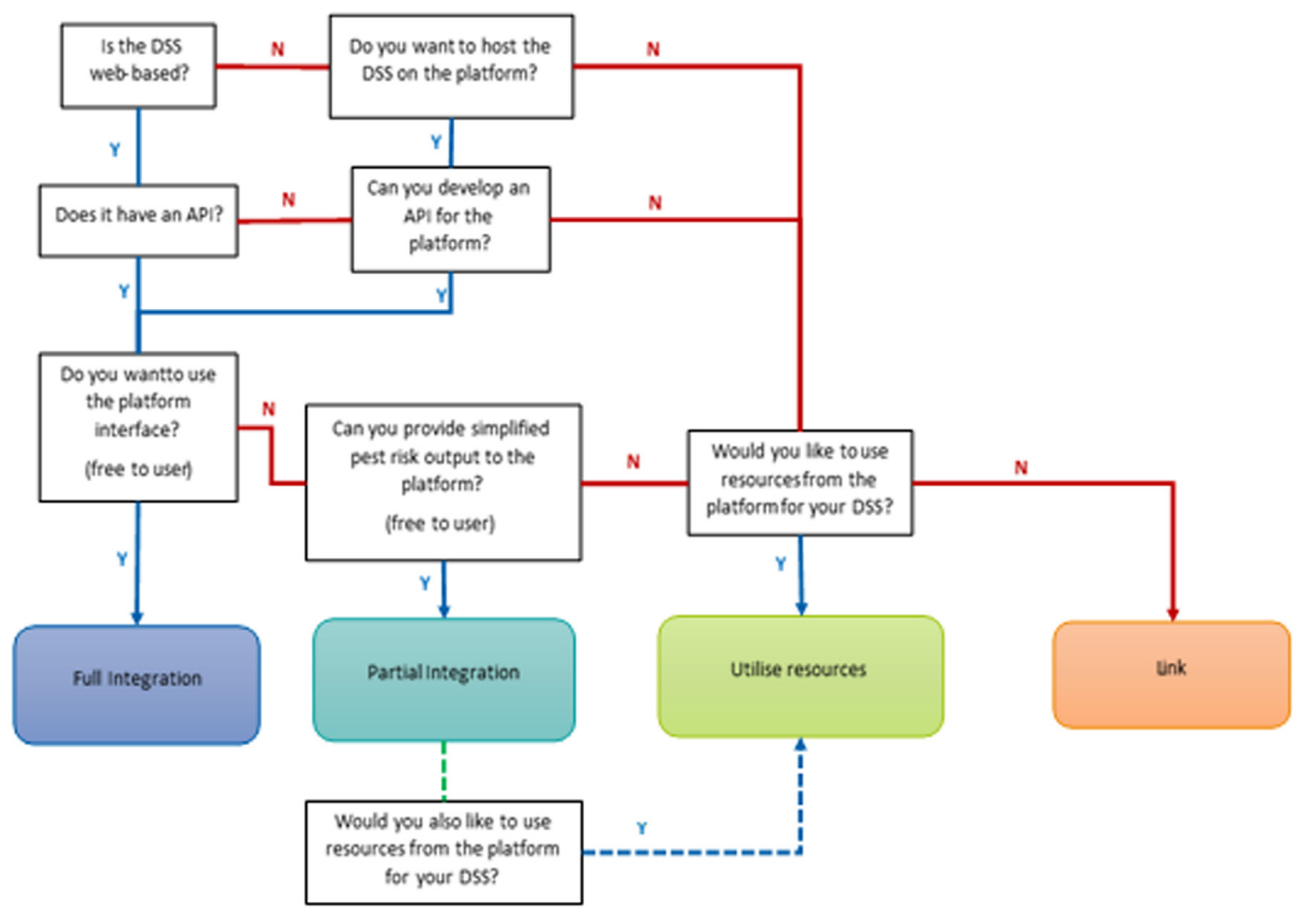
Summary of methods by which DSS can interact with the IPM Decisions Platform.

An individual DSS will interact with the Platform by one or more of the four modes above. Fully integrated DSS will utilize resources from the Platform. Partially integrated DSS may choose to utilize resources. Figure 2 provides a decision tree for selecting the appropriate integration option for a DSS.

**Full integration of DSS into the IPM Decisions platform**

Full integration embeds the DSS within the platform, using the platform user interface and the DSS is only accessed by users via the platform. In this case, the DSS must comply with the platform data standards. The user sees the DSS outputs within the DSS Use Dashboard, and has access to the background information detailing the models and origin of the systems. This is most likely to be appropriate to DSS that are in the public domain, which may not be web-based currently or where the DSS developers do not have resources to develop a sophisticated interface for the DSS and promote its use to a large user group. The Platform will manage the inputs and outputs.

**Case example: full integration of four pest forecast models**

In collaboration with the University of Warwick (UoW), four pest forecasting models developed in the UK ( Table 1) were fully integrated into the IPM Decisions platform. The models were originally developed in the 1990s and were part of the MORPH software ( Collier, 2007), which had become very dated and its compatibility with future upgrades of Windows was in doubt. The process of reviewing the existing MORPH models, converting them into compatible formats for integration, development of the associated metadata, integration into the IPM Decisions Platform, testing, and publication took around 4 person months across all parties. Each model uses a combination of within season climate data provided by the platform weather data APIs and historic soil temperature data, uploaded to the platform for a set of UK locations. These models require a source of forecast soil temperature data to predict the phenology of the pests, but as no open access database of soil temperature is currently available the models use a static dataset containing five year mean hourly soil temperature at 6cm depth, collected at ten locations between 2017–2022. Users select the most appropriate locations for their selected farm location. Work has subsequently been completed to extend access to these models to users in Belgium. Through provision of additional historic soil temperature data.

**Table 1.** Summary of the four MORPH DSS integrated into the IPM Decisions platform in collaboration with the University of Warwick.

DSS name	Targeted pest	Relevant crop(s)	DSS Purpose	References
**Cabbage root fly (Warwick HRI)**	Cabbage root Fly *Delia radicum*	Brassica family	The model predicts the timing of adult emergence and egg-laying throughout the year, enabling users to undertake targeted monitoring and/or mitigation actions to reduce the risk of damage to the crops.	Collier & Finch, 1983a Collier & Finch, 1983b Collier & Finch, 1986 Finch & Collier, 1983 Finch & Collier, 1985 Finch *et al.*, 1986 Phelps *et al.*, 1993
**Pollen Beetle (Warwick HRI)**	Pollen beetle *Meligethes aeneus/ Brassicogethes aeneus*	Cauliflower, Broccoli	The model predicts the timing of spring emergence of adult beetles, egg laying and then the emergence of a new (summer) generation of adults ready to disperse, followed by their dispersal.	Phelps *et al.*, 1993
**Carrot fly (Warwick HRI)**	Carrot fly *Psila rosae/ Chamaepsila rosae*	Carrot, parsnip, celeriac, parsley	The model predicts the timing of adult emergence and egg-laying throughout the year, enabling users to undertake targeted monitoring and/or mitigation actions to reduce the risk of damage to the crop.	Phelps *et al.*, 1993 Collier *et al.*, 1994 Collier & Finch, 1996
**Large narcissus fly model (Warwick HRI)**	Narcissus bulb fly *Merodon equestris*	Narcissus species (daffodils)	The model predicts the timing of adult emergence, egg laying, and egg hatching, enabling users to undertake targeted monitoring and/or mitigation actions to reduce the risk of damage to the crop.	Collier & Finch, 1992 Phelps *et al.*, 1993

**Partial integration of DSS into the IPM Decisions platform**

Partial integration is more appropriate for DSS which already have a well-developed user interface and want to reach a larger group of users. The DSS may be free to use or a commercial system. The DSS would provide a risk output in the format of the IPM Decisions Platform, increasing visibility of the system across users, who would be signposted to the full version which may sit behind an external paywall.

**Case example: integration with Crop Protection Online, Denmark**

In Denmark, a series of DSS are available on the Crop Protection Online ( CPO, 2025), managed by Aarhus University and SEGES. CPO is a commercial service, offering a range of resources supporting IPM through existing user interfaces with an established client base. As such, partial integration of DSS into the IPM Decisions platform was the most appropriate route to widen awareness and access. Simplified versions of a selection of DSS for wheat and/or barley disease management were developed and linked via API to the platform. For these models, the user is required to provide data on the susceptibility of their crop varieties to the disease (resistant or susceptible), a disease observation (percentage of plants currently showing infection), and the growth stage of the crop when the observation was made. Based on this information, the user is provided with a forecast risk and associated guidance. In this case, partial integration has enabled platform users outside of Denmark to access these established DSS without any commercial compromise from the original DSS providers.

**External utilization of IPM Decisions resources**

The ‘Utilise resources’ option is appropriate for external DSS developers wishing to make use of the free resources available through the Platform, including access to the ‘open access’ DSS integrated within the Platform (which may be complementary to their own DSS or be modified for a different region/purpose), and access to weather data.

**Case example: integration with VIPS, Norway**

The VIPS ( VIPS, 2025) platform has been running in Norway for more than 20 years, forecasting risk levels for pests at locations around weather stations across the country. All the VIPS models are implemented within the system, and the majority have also been fully integrated by API into the IPM Decision platform. DSS available within the IPM Decisions platform can also be implemented in VIPS, such as the saddle gall midge model ( Rowley *et al.*, 2017). This DSS was set up in VIPS by writing client code that utilizes the metadata for the saddle gall midge model to automatically display the correct input data form fields and to identify which weather data are required. When the model is run it selects the appropriate weather data sources and compiles this with the input data into the format required. This information is then sent to the web service in the IPM Decisions system, which runs the model and returns the results in a common format. This is then rendered onto charts as required for the VIPS system. This process enables external DSS providers to run IPM Decisions DSS using alternative weather data, potentially at a higher resolution that the open-source data used in the original platform, or to use IPM Decisions weather data. External providers utilising resources in this way are required to cite the platform as the source of the risk forecast.

**Linked DSS with the IPM Decisions platform**

Linked DSS are made accessible through the platform External Link DSS Dashboard. Here, users can find external DSS, and follow links to those of interest. Links may be appropriate either where the DSS type does not align with the platform data standards or suit the interface structure, or where DSS are more strategic rather than tactical in the types of decision supported.

**Case example: BEST4SOIL**

Farmers’ incomes depend to a large extent on the state of their soil health. However, information to help tackle the specific issues affecting soils can be hard to come by. As part of the EU-funded BEST4SOIL project (2018) two online DSS were developed supporting management of nematodes and soil-borne pathogens. These systems are highly relevant for inclusion in the IPM Decisions platform, however they do not operate as risk forecast systems, but instead provide details of the relative tolerance/resistance of crops and varieties against key soil-borne pests. To support access to these DSS, a link to each is provided within the dedicated ‘External Link DSS Dashboard’, which enables users to select, review and transfer to an external platform.

**DSS Innovation**

A new service – the IPM Decisions Factory – has been created, implemented and hosted on the scientific framework OpenAlea ( Pradal *et al*., 2008). It allows users to transform any OpenAlea node or Python function annotated with the required inputs and outputs, into an IPM-Decision DSS ready to be deployed on the platform. The created DSS consists of an auto-generated docker file hosting a web service fully compatible with IPM-Decisions, and an auto-generated *json* file that allows registering the new model in the IPM-Decisions DSS catalogue. A demonstration example of the DSS integration use has been released on GitHub, in the form of a jupyter notebook ( IPM Decisions, 2023b). The IPM Decisions Factory enables DSS researchers and developers to advance, combine and create DSS, which are then readily integrated into the IPM Decisions platform. This ensures that new DSS have access to required weather data and can be made readily accessible to across Europe, for validation and use. OpenAlea.EpyMix ( Levionnois *et al*., 2023) is a model describing canopy growth and epidemic dynamics on species mixture that has been integrated into the IPM Decision platform to understand how weather data, provided by the platform, and wheat-based crop mixtures are a promising strategy to improve disease management.

***DSS Metadata***

Irrespective of the route to integration, DSS developers are required to provide a description (metadata) of the models and associated parameters within the DSS, input and output lists with units and descriptions, and references. The amount of detail required on these points varies with the level of integration. If access to DSS or DSS outputs via web API are required, developers will need to provide support for platform requests in standard format, and the ability to modify parameters of the DSS to comply with the platform standards. DSS developed through the IPM Decisions Factory will include all required metadata. A DSS API has been developed, establishing a standard format for the integration of DSS into the platform. This approach ensures that new DSS can be readily integrated without impacting the function of existing DSS or the platform user interface. Source code for the DSS services and climate services APIs are available in GitHub ( Skog, 2024). To enable effective completion of DSS Metadata by prospective DSS providers, a DSS Metadata file editor has also been developed, along with a supporting guide ( IPM Decisions, 2023a).

### Consulting the IPM Decisions platform

The home page of the platform provides introductory information about resources for the three key target groups; farmers and advisors, researchers and developers. Summary risk maps are available on the home page, displaying a selection of upcoming pest risks. While these are restricted to fixed assumptions and not robust for all potential users, they alert users to potential risks in their region that may require further attention. To obtain location and crop specific risk forecasts, users register an account and set up a farm location. The ‘Farm Management’ dashboard enables users to set up multiple farm locations anywhere in Europe and add DSS for selected crop/pest combinations. DSS developed in ‘other’ countries are available for farmers and advisors all over Europe, with the option to restrict selection to regionally relevant systems. Farm set up also connects the farm location to the relevant weather data sources needed to run DSS. If users have their own weather station with data stored in a cloud based database, and linked to the IPM Decisions platform (currently available for Metos, MeteoBot, and Fruitweb) this data can also be used.

***Consulting IPM DSS integrated into the IPM Decisions platform***

Once a farmer or advisor has set up their farm on the platform, and set the relevant parameters for each system, the ‘DSS USE’ Dashboard provides farmers and their advisors with an overview of the risk situation for the crop/pest interactions selected. From there, specific risks can be investigated, underlying parameters can be updated, and additional information is available to support interpretation and application of the outputs. The user needs to review the default parameters for each system and adjust them according to their farm conditions. This may include adding details on crop drilling date, growth stages, plant protection product (PPP) applications, and other agronomic details relevant to decision tool algorithms. This information may require updating throughout the season to generate appropriate risk outputs and guidance.

***Comparing and adapting IPM DSS within the IPM Decisions platform***

The IPM Decisions platform enables users to compare and adapt systems and to review the impact of different locations and/or parameter adjustments on the forecast risk. The ‘DSS Comparison Dashboard’ facilitates the comparison of any combination of selected, active DSS on the user’s account. This can be done for the same or different systems and can be used to look in more detail at the relative risk of a given crop/pest combination at multiple locations. The Comparison Dashboard can also be used to compare the outcomes from different DSS, where systems targeting the same pest have been developed in different regions. This ability to compare alternative models can help reinforce risk alerts or highlight where pest development rates or emergence may be regionally diverse. In addition to within year comparisons, for some models it is possible to compare current year forecasts with forecasts from previous year. This helps frame the risk within the context of previous user experience. The extent to which the historic data matches the user’s own experience will influence the extent to which the current year forecasts are incorporated into management decisions.

The ’DSS Adaptation Dashboard’ was primarily designed for advanced users and researchers, looking to assess and adapt DSS for new regions. This adaptation process can enable fast-track access and validation of established systems, without the need for extensive model redevelopment. The extent to which a DSS model can be adapted is dependent on whether the original developer agreed to make the underlying parameters public and accessible. In the Adaptation Dashboard, these parameters can be adjusted where existing evidence justifies it, and the resulting new version can be reviewed and added back into the DSS USE dashboard. This enables the user to consult the adapted version throughout the season and ideally collect appropriate data to enable validation in the new region; after which it can be formally reviewed, integrated and made available to a wider group of platform users.

### Platform disclaimer

Users registering an account on the IPM Decisions platform are required to accept the platform Terms and Conditions ( IPM Decisions, 2023c), and are made alerted that the platform makes DSS available from external DSS developers. The 'gold standard' for DSS is that they have been created by reputable DSS developers and researchers, tested, validated, and widely used in practice over many sites and seasons, and the results published in peer reviewed journals to demonstrate useful predictive value in the countries in which they are recommended for use. Few DSS meet all these criteria. IPM Decisions aims to make a wide range of DSS available for testing and use through a free access web-based integration platform. Therefore, it is not a requirement that all these criteria are met for a DSS to be available through this Platform. Instead, the IPM Decisions management take reasonable care that the DSS are from reputable developers and institutions and further information is provided about each DSS, to enable an informed choice about use. The DSS can be used to assist (not replace) experienced crop managers in decision making process, taking into account all relevant local pest risk factors. Farmers and advisers should not use the DSS to guide decisions on substantial areas of crop until they have gained experience in their use and have found the risk estimates reliable. Erroneous risk estimates can occur, particularly if conditions are outside those experienced during DSS development and testing. Farmer groups and research organisations are encouraged to test the DSS in the field, amend the DSS to suit local conditions if necessary, and share their experiences.

### The validation and evaluation of IPM DSS

IPM DSS are more likely to be used if there is evidence of economic returns. Testing a DSS usually involves comparing predictions of pest risk against observational data of pest prevalence ( Andersson *et al*, 2022; Jørgensen *et al.*, 2020; Jørgensen *et al*., 2021). Predictive value does not, however, necessarily translate into economic benefits. To enable economic analysis, methods have been developed and tested on data sets in which pest observations were collected across several sites and seasons, including field trials testing DSS for septoria, grape downy mildew and apple scab. ( Holst, 2022a; Holst, 2022b; Holst, 2022c; Holst, 2022d; Holst, 2022e; Holst & Donner, 2022a; Holst & Donner, 2022b). It was found that, in general, data from field trials conducted for other purposes (e.g. pesticide efficacy trials) are not ideal for the validation of DSS. Literature studies revealed that field trials purposely designed to test DSS resulted in better evidence for DSS validity. Tools were developed to assess the benefits of DSS, both economically and environmentally (in terms of reduced pesticide use) and found that use of DSS offers benefits on both accounts compared to fixed spraying schedules ( Helps *et al.*, 2024).

The value of a DSS is defined as the economic and/or environmental benefit derived from using a DSS over standard practice. A framework for describing how these two types of benefit can be quantified has been developed for various types of DSS and for various formats of validation data. Specifically, the methods consider the situations where DSS inform (i) the number of sprays (ii) the total dose of pesticides (iii) the timing of the onset of spraying, and (iv) the risk of a pest outbreak. To be meaningful, estimates of the expected value of DSS should be accompanied by a quantification of the likely variation in value, hence allowing the user to make a risk-based assessment. In brief, the procedure is as follows:

1. Estimate the distribution of pest intensity in untreated fields, *g*( *x*);
2. For each experimental site, generate pesticide application programmes guided by the DSS that you want to test;
3. Estimate the relationship between the amount of yield lost given a particular pest intensity and any application programme;
4. Sampling from the pest intensity distribution in (1), estimate the cost of using a standard application programme or a DSS-guided application programme.

By providing a framework to estimate the economic value of DSS in different regions where pest conditions may be different, users can be provided with greater knowledge about the potential benefits and risks of any given DSS. While there may be many DSS available for a particular pest/cropping system, most of them are tested and recommended only in the country or countries where they have been developed, despite those pest/cropping systems existing elsewhere. Enabling users to quantify the potential risks and benefits of such DSS in their own region allows testing of DSS to check if they have value for use beyond their current bounds. Full details on the methods to self-validate and evaluate DSS have been published by Helps *et al.* (2024).

To determine the wider value of using DSS in European agriculture, a high level comparison was made between crop production where these systems have been implemented, and those where traditional management practices are used. The resulting differences in yield, cost of production and treatment frequency index (TFI) were used to evaluate the overall value of implementing DSS. A meta-analysis, undertaken in IPMWORKS determined the effect of using DSS on treatment frequency index (TFI), yield and disease severity ( Furiosi *et al*., 2025). The TFI is calculated by dividing the total quantity of active ingredients used for each crop by the standard dose associated with each active ingredient ( Gravesen, 2003; Kudsk, 1989). The meta-analysis study used data from 10 papers for wheat, 31 for potatoes and 24 for grapes (from a total number of 769 studies over 27 countries covering the period from 1983 to 2020). It focused on those papers where there was a comparison between the efficacy in crop protection under standard agricultural practice and implementation of DSS, with data given on treatment numbers and yield impacts (for wheat and potatoes). Data was collected on total weight of pesticide active substance used at the national/European level and cost of pesticide applications at the field level, for wheat, potatoes and grapes. This information was used to estimate the potential scale of impact on production and costs if pesticide usage was reduced through increase uptake of the DSS on farm. Effectively, this created a partial budget calculation to understand the impact of change on the business (at a per hectare level) and then scaled up to country and EU level.

The use of DSS provides growers with the opportunity to reduce their pesticide usage, which when scaled up to a national or EU-level, results in significant estimated savings both in terms of pesticides required, as well as total cost. This demonstrates the value that the IPM Decisions Platform has to European agriculture by providing access to a variety of DSS for the major outdoor crops. An increase of application of IPM DSS by 25–50% in wheat, potatoes and grapes would make a substantial contribution towards the European Commission’s targets to reduce pesticide use, and risks associated with pesticide use ( Webb & Wynn, 2023). A number of simplifying assumptions had to be made for this analysis, which found that when scaled up to the European level (EU 27), it is estimated that 25% additional uptake of DSS would result in total pesticide usage in wheat reducing by 1,800 tonnes, in potatoes reducing by 1,700 tonnes and in grapes reducing by 5,700 tonnes. The biggest reduction is seen when an additional 50% of the crop area is under DSS, as these values increase to 3,600 tonnes for wheat, 3,400 tonnes for potatoes and 11,500 tonnes for grapes. This is equivalent to pesticide usage being reduced by an estimated 24% across the three crops, which results in a 28% reduction in pesticide costs, worth €4,000 million a year.

### IPM DSS case study – using the Septoria Humidity Model in Sweden

In Sweden, and across Europe, severe infestations of leaf blotch disease of wheat can be caused by septoria tritici blotch ( *Zymoseptoria tritici*), driven by weather and host susceptibility ( Jalli *et al.*, 2020). This disease is often managed by routine growth stage-based spray programmes applied either as preventative measures at vulnerable growth stages or based on observations of early disease ( Zadoks, 1985). Early-stage disease severity assessments are thought to have little correlation with crop damage ( Jalli *et al*., 2020; Paveley *et al*., 1997). Later disease assessments conducted during flowering are better correlated with yield losses but are too late to influence the timing of fungicide applications ( Andersson *et al*., 2022). Using weather-based risk models to estimate future disease development at a time when fungicide applications are still effective show the greatest potential to optimize fungicide inputs ( Andersson *et al*., 2022; Burke & Dunne, 2008; Jørgensen & Hagelskjær, 2003; Jørgensen *et al*., 2020).

In a comparison run as part of the IPMWORKS in-field comparison activities ( Francis *et al.*, 2025), a DSS from Crop Protection Online (CPO) previously validated in Nordic and Baltic countries ( Jørgensen *et al*., 2020), was used to guide the timing of applications of fungicides in seven fields within different regions of Sweden. This DSS has also been integrated with the IPM Decisions Platform, making it accessible across Europe. The trials were part of the annual fungicide reference trials conducted by the Swedish Board of Agriculture. The specific aim of the DSS treatments was to demonstrate the use of a DSS to time fungicide applications as part of management of wheat diseases across seven farms. The IPM DSS strategy was compared with several other growth stage-based treatment applications, and data was collected to determine the effectiveness of each approach in managing priority wheat diseases. This comparison was completed in collaboration with the Swedish University of Agricultural Sciences (SLU). In each of seven trials, two disease management approaches were implemented and compared with an untreated control. Approach 1: Four alternative fungicide application programmes were implemented based on crop development stages. Approach 2: Under the ‘DSS led’ approach, fungicides were applied according to DSS output timings. In all cases, the DSS approach Treatment Frequency Index (TFI) was either zero or 0.5, equivalent to the untreated/lowest development stage approaches. Partial economic analysis revealed that the most intensive treatment (treatment 5, TFI 1.2) was consistently the least profitable approach. Consultation of the DSS provided reliable guidance on disease management, supporting effective treatment while minimising fungicide applications where septoria was the key concern.

Combining the consultation of a decision support system with a farmer or advisors knowledge of disease epidemics shows great potential in optimising fungicide programmes. Not applying fungicides in a very low disease pressure season is a difficult decision to make; a low risk rating from a DSS can give growers the confidence not to apply fungicides in these scenarios. In slightly higher disease risk scenarios where the value of a fungicide is debated, a DSS can again support a single application of fungicide to optimise yield. The examples demonstrated here highlight scenarios where a DSS can support the reduction of fungicide usage where not deemed necessary, saving the grower money and reducing the impact of pesticides on the environment. Full results of this study, along with further IPMWORKS in field comparisons of IPM approaches are available in Francis *et al.* (2025).

### Incentives and barriers to IPM DSS uptake

On a given farm there are many potential pest threats on a range of crops. DSS for each crop/pest combination may have been developed, but access is currently through multiple platforms, often limited by region, provider, crop, pest, or language. The analysis of survey responses collected in multi-actor workshops has identified barriers to uptake of IPM DSS, and measures to overcome them. Some elements are common across Europe, others are region or sector specific. A detailed assessment of overcoming barriers to uptake of IPM DSS is provided by Marinko *et al*. ( 2025a; 2025b) and Akaka *et al.* (2024), which revealed region-specific barriers to DSS adoption among both farmers and advisors. Although many DSS are potentially useful in many more countries, validation work is essential to build trust and engagement among potential users. Details of the analyses are available in Marinko *et al.*, 2025a; Marinko *et al.*, 2025b, and Akaka *et al.*, 2024, the main findings were:

- Across Europe, farmers that were less inclined to use IPM DSS exhibited a lack of trust in DSS and held the perception that they lack sufficient IT knowledge.
- A common barrier to the use of DSS among farm advisors was poor access to reliable, independent information about DSS.
- Among farm advisors across Europe, there was a high level of understanding of the purpose of IPM DSS, and most reported that IPM DSS are a complement to, and not a substitute for, their work.

Further analysis of the socioeconomic aspects of barriers to adoption highlighted that platforms need to be easy to use, and that building experience with simple systems build confidence and promotes wider adoption of DSS ( Akaka *et al*., 2024). The availability of DSS differs significantly between countries across Europe. In some regions, long term investment in the development of IPM DSS has generated a range of systems and associated public and/or or commercial platforms, while elsewhere access is highly restricted and limited to commercial enterprises. While commercial platforms can offer advanced, reliable services, payment walls represent an important barrier to access and uptake, and the willingness to pay for IPM DSS is influenced by wider factors beyond the services offered, notably experience using IPM DSS can be critical in the decision to pay for further access ( Akaka *et al*., 2024).

Farm advisors play an important role in promoting and increasing the use of DSS among farmers ( Rose *et al*., 2016). Communication between the farm advisors and farmers is crucial to successfully increase the use of DSS in agriculture, as it provides farmers with information on the reliability of DSS from someone they trust ( Kuehne *et al.*, 2019). The IPM Decisions Platform provides an open access first point of contact for farm advisors looking to engage with DSS, however there is a need to organise educational or demonstration workshops as well as including DSS training in agricultural schools and universities. The analysis also highlighted the need for national support to facilitate the longevity of open access decision support platforms, as well as improving high-speed internet access on farms across Europe. To overcome the identified barriers to IPM DSS adoption, 14 measures for DSS developers were proposed ( Table 2). The listed measures can be used as a checklist for DSS developers to ensure that newly developed DSS meet certain quality and usability standards, thus facilitating the uptake and use of DSS and supporting a greater impact of IPM DSS in Europe.

**Table 2.** Summary actions for increasing the impact of decision support systems for IPM, adapted from Marinko *et al.*, 2025b.

Number	Action
1	Provide clearly structured outputs from DSS that allow users to obtain an understandable (not too general or very detailed) description of the crop damage risk assessment and proposed mitigation measure
2	Provide access to validation data or to publications describing the validation results
3	Provide instruction in text or video form
4	Involved farmers, farm advisors, and researchers in the further development or validation of tools and take their advice and suggestions for improvement into account.
5	Offer a free, limited trial version if the DSS is chargeable.
6	Ensure that the user interface is easy to use
7	Minimising the manual inputting of data that can be automatically obtained (e.g. weather data).
8	Allow the input of field observation data without internet access (for DSS running on mobile phones and tablets)
9	Translate DSS into the local language of providing a translation into a language commonly spoken/ understood by users in the country.
10	Integrate DSS with existing regional platforms
11	Organise DSS workshops with farm advisors and researchers and offer them the opportunity to run or collaborate in DSS workshops for farmers
12	Encourage users to share positive experiences with other farmers, farm advisors, or researchers
13	Provide transparent descriptions of the characteristics and requirements of the DSS so that users can compare it with other DSS and choose the one that suits them best
14	Develop DSS for underrepresented sectors and their needs (e.g. viticulture, vegetables, hops, olives and other fruit)

***Access to information on available IPM DSS***

Information about IPM DSS is often scattered across the websites of different providers and DSS developers. The lack of standardized descriptions makes it even more difficult for the user to compare DSS from different DSS developers. To overcome this, Marinko *et al*. (2024) developed the IPM DSS typology, which provides a standardized framework for the description of IPM DSS. The IPM DSS typology is implemented in the free web-based tool IPM Adviser ( https://ipmadviser.ijs.si/, accessed on 26. 6. 2025). The IPM Adviser currently includes 79 DSS, each characterized by more than 50 structural and performance characteristics. By providing reliable, comparable information in one place, the IPM Adviser helps farmers and farm advisors overcome one of the main barriers to wider adoption — limited access to clear, consistent information on the available IPM DSS ( Marinko *et al*., 2025a).

## Current and future contribution of IPM DSS to sustainable use of pesticides

The SUD aims to achieve a sustainable use of pesticides in the EU; reducing the risks and impacts of pesticide use on human health and the environment. It forms part of the EU legal framework covering pesticides and their use. By promoting the use of IPM, the SUD supports the achievement of the targets set out in the EU’s Farm to Fork Strategy ( European Commission, 2020). IPM Decisions was planned and delivered in consultation with national authorities, represented as project participants, responsible for implementation of the SUD. The Danish Environmental Agency (DEA) and UK Health and Safety Executive (HSE) were partners within IPM Decisions consortium. Their role was to provide insight on the role of DSS in regulation and to inform regulatory colleagues in other EU member states about the IPM Decisions platform. This included updates as part of regular meetings of the EU working group of experts on Sustainable Use of Pesticides within the Directorate-General for Health and Food Safety (DG SANTE), responsible for EU policy on food safety and health and for monitoring the implementation of related laws. In 2023, IPM Decisions collaborated with the IPMWORKS consortium in developing policy recommendations in support of the debate on the proposed SUR ( IPMWORKS consortium, 2023). IPM Decisions presented three policy recommendations to support access to and consultation of IPM DSS in Europe, in line with wider efforts to increase application of digital services and in support of the SUD and Green Deal objectives; these were to i) support research and development of novel IPM DSS, ii) address technological and socioeconomic barriers to IPM DSS consultation, and iii) support wider demonstration of IPM DSS in practice.

### Support research and development of novel IPM DSS

Development of IPM DSS involves large amounts of data and analysis, as well as integration into a suitable platform to make it accessible. A more coordinated approach to DSS innovation across Europe, with clear guidelines on the requirements and standards for DSS provision in agriculture, would both improve trust in DSS performance and drive innovation.

- **Coordinated DSS development:** Development of novel DSS is disjointed across Europe, leading to high levels of diversity in approach and delivery, including in the design of graphical user interface. Greater integration of IPM DSS development and applications should be included in educational curriculums, especially in agricultural courses at university and agricultural schools.
- **Account for risk aversion:** The perceived level of risk represented by a given pest-crop interaction can be influenced by a range of factors, both directly and indirectly linked to the likelihood of a damaging infestation. The extent of pre-decision risk aversion will influence interpretation of DSS outputs. Further research and support is needed to engage decision makers, so that decision making processes incorporate the effect of individual and/or group risk aversion in pest management decisions.
- **IPM DSS Validation:** Novel DSS models, or models that have been translated and/or adapted to novel regions, need to be independently validated against clear standards. DSS providers should be bound to provide evidence of the extent to which DSS outputs are valid at a given location, and what assumptions or limitations should be considered when interpreting DSS outputs.
- **Quantify the benefits of DSS:** Development of novel DSS often focuses on the generation and testing of the algorithms required to make risk predictions. Research is required to further evaluate the economic and environmental value of DSS, as well as identifying critical pest/crop combinations where high value returns can be achieved.

***Address technological and socioeconomic barriers to farmers and advisors IPM DSS consultation***

A number of technological and socioeconomic barriers to the uptake of IPM DSS have been identified for both farmers and farm advisors.

- **Digital infrastructure:** Many farmers and advisors do not have consistent access to information and communications technology (ICT) infrastructures required to reliably engage with online systems, notably access to internet connection across farming landscapes. Support for improved digital infrastructure, especially for improving rural broadband, would improve uptake of online DSS services.
- **ICT and DSS training:** Many farmers and farm advisors perceive their ICT skills as in need of improvement to better engage with online systems. Support for wider training in the access to, consultation and interpretation of IPM DSS would improve uptake of online DSS services.
- **Targeted DSS development:** Encouraging/subsidising the development of DSS in regions and for crops where there are few or no existing systems (e.g. olives, vines, hops in Southern and Central Europe).
- **Subsidising DSS subscriptions:** Open access to DSS system at an introductory level should be subsidised to ensure widespread use and resulting societal benefits. Support for access to more advanced, bespoke systems would also improve uptake, but this would be further enhanced where users have had previous experience with open systems.

***Support wider demonstration of IPM DSS in practice to farmers and advisors***

In all parts of Europe, farmers and advisors change practices based on access to reliable information on a novel approaches. For IPM DSS, a lack of trust in the outputs from risk forecast models was identified as a key barrier to uptake. Engagement with these groups highlighted that marketing promoting generalised benefits of DSS that do not provide transparent information on the validity or limitations of their application may be counterproductive to building trust. While consultation of DSS from other regions may be considered as part of decision-making processes, it is unlikely to lead to sustained changes in practice without regional validation and demonstration.

- **IPM DSS Demonstrations:** To build trust in the use of IPM DSS, independent demonstration of the application and impact of DSS are required at a regional scale, including summaries of the short- and long-term impact on production, and profitability of the crop.

## Conclusion

The IPM Decisions platform supports farmers and advisors by bringing together DSS from multiple sources in a consistent format, enabling rapid consultation of the future risks posed by pests. By reducing the ambiguity around the potential risks to the crop, farmers and advisors can make better informed decisions as part of a holistic IPM approach. For IPM DSS researchers and developers, the platform provides resources for integration, adapting, and running DSS from across Europe. Researchers benefit from access to a Europe wide end point for forecast models, and Developers can utilise the platform infrastructure to expand the range and scale of systems they can use. By improving open access and consultation of DSS, the IPM Decisions platform supports national initiatives to promote holistic IPM approaches, and improve sustainable crop protection across Europe.

## Disclaimer

The views expressed in this article are those of the author(s). Publication in Open Research Europe does not imply endorsement of the European Commission.

## Ethics and consent statement

This article summarizes results obtained in the Horizon 2020 IPM Decisions project. Ethical approval and consent were not required for this article. When necessary for individual studies and survey, ethical approval was received, and detailed in associated publications.

## Data Availability

No data is associated with this article. When data was collected as part of individual studies and survey, data is detailed in associated publications.
