# Supplementary material for: IPM Decisions Platform – a Pan-European online platform hosting decision support systems and associated resources for integrated pest management
Source: Open Res Eur. 2025 Oct 16;5:320. [Version 1] doi: 10.12688/openreseurope.21411.1 (PMC12605576; doi:10.12688/openreseurope.21411.1)
Supplement: Peer review history

# Reviewer report: Reviewer response for version 1

**DOI:** 10.21956/openreseurope.23160.r62430

**Published:** 2025-11-11

**Authors:** Daniel J. Leybourne

**Affiliations:** 1 The Agri-Food and Biosciences Institute, Liverpool, England, UK

This open letter describes the IPM Decisions platform, a platform that aims to integrate DSS across Europe into a simplified database that can support DSS use and uptake. The platform itself is user-friendly and has the potential to develop into a cornerstone of sustainable agriculture across Europe. This letter is written well and introduces the system and wider context sufficiently. I have no major comments but have provided some minor feedback below.

 The title could better reflect the contents of the manuscript – one could read the title and expect to find all the associated DSS detailed, whereas what is contained is an introduction to the wider project and an overview of the system itself. This could easily be addressed by changing the title to: Introducing the IPM Decision Platform – a pan-European online platform for hosting decision support systems and associated resources”. A summary of the current integrated DSS, grouped into “fully integrated”, “partially integrated” etc. would also be useful / would address this point.

  

- The background could be developed some more, potentially through greater introduction of sustainable agriculture and the impact pests/disease have. Currently the manuscript starts by introducing DSS along the lines of the IPM Decisions / IPMWORKS projects with limited context.
  - The need for DSS could be better detailed by providing a brief introduction to IPM  in general. This could be focused around the IPM pyramid and the eight IPM principles - these principles are used later in the introduction, but not described/introduced as a cohesive scheme. This could be done by moving some of the text in the “Validation and evaluation of DSS” section into the intro section, as the background context on DSS and their purpose is detailed sufficiently in this subsequent section.
    - The authors introduce the IPM pyramid/principles but don’t reference Barzman et al., 2015 where these principles were introduced (https://link.springer.com/article/10.1007/s13593-015-0327-9)
  - The authors can then use the IPM principles to detail how DSS can contribute here to develop sustainable IPM
- The concept behind the IPM Decisions platform is well explained, and I think the platform itself will represent a step-change in the delivery of DSS. As DSS uptake is usually restricted by i) difficulty in accessing DSS; ii) high expertise required to run some DSS (e.g., code-based systems). The platform described here is an avenue through which these restrictions can be addressed and DSS uptake and use increased.
- A key strength of the platform is the consistent engagement with stakeholders when designing and developing the system, this will help ensure the system works for the end-user. This is an incredibly important aspect that is often overlooked.
- Availability of the system (including source code) in multiple languages will also support uptake amongst stakeholders.
- Integration with meteorological APIs will support on-the-fly use of the platform and the associated DSS, removing the need for the user to provide this information themselves and removing the risk of ineffective DSS use; often this meteorological data underpins the functioning of DSS and therefore needs to be accurate and reliable.
  - The ranking of the available weather data helps to address any issues associated with low quality/missing data, and the associated warning will help users balance any resulting decisions against these caveats.
- The introduction and description of the different model types is good. The authors could expand these descriptions by providing examples of specific DSS for each type if available.
- Is Figure 2 needed?
- The four integration options are sensible, and should increase uptake and integration with research-led development of DSS (which usually lack a useable GUI) and government/industry developed DSS (which usually have a GUI, but could be better signposted). The case study of the Warwick examples shows that the IPM Decisions platform can work for the integration of older models.
- Many of these systems will have been developed under specific agro-environment conditions (e.g., the examples in Table 1 are all UK-based) and might not be directly transferable to another country without on-the-ground testing and validation. The “DSS Adaptation Dashboard” starts to address this, by offering a way for researchers to test + validate DSS in other regions.
- The “Incentives and barriers to IPM DSS uptake” section could be placed in the introduction, this would allow the authors to highlight a key “challenge” of DSS use and present the IPM Decisions platform as a solution for this.
- The 14 actions for increasing DSS use (Table 2) is a sensible list that will hopefully support the development of future DSS.
- There are some very minor spelling errors, e.g., number 4 in Table 2 should read “involve” not “involved”. I recommend a final proofread of the manuscript.

Where applicable, are recommendations and next steps explained clearly for others to follow? (Please consider whether others in the research community would be able to implement guidelines or recommendations and/or constructively engage in the debate)

Yes

Does the article adequately reference differing views and opinions?

Yes

Are all factual statements correct, and are statements and arguments made adequately supported by citations?

Yes

Is the rationale for the Open Letter provided in sufficient detail? (Please consider whether existing challenges in the field are outlined clearly and whether the purpose of the letter is explained)

Yes

Is the Open Letter written in accessible language? (Please consider whether all subject-specific terms, concepts and abbreviations are explained)

Yes

Reviewer Expertise:

Sustainable agriculture; the sustainable management of arthropod pests and vector-borne disease in agro-ecosystems

I confirm that I have read this submission and believe that I have an appropriate level of expertise to confirm that it is of an acceptable scientific standard.

# Reviewer report: Reviewer response for version 1

**DOI:** 10.21956/openreseurope.23160.r62434

**Published:** 2025-11-06

**Authors:** Daren S. Mueller, Laura Iles

**Affiliations:** 1 Plant Pathology, Entomology, and Microbiology, Iowa State University, Ames, IA, USA

The paper, which is an Open Letter introducing the IPM Decisions Platform, focuses primarily on the platform's architecture, development process, and strategic goals. It provides excellent detail on how Decision Support Systems (DSSs) are integrated to improve adoption of DSSs. The article is very well written and provides a wealth of information regarding the development of the IPM Decisions Platform.

 Often with large, complicated projects we do not get a summary of the reasoning behind the project, the pitfalls, constraints and the successes. We do often get specific research papers about portions of the project (like one disease system) where the infrastructure behind it is acknowledged, but we do not get papers focusing on the infrastructure. This paper will be valuable to others doing similar work across the world and serve as a framework for how to share the varied aspects of such an undertaking.

 Here are a few things to consider for improving the article.

1. "IPM Decisions Platform - a Pan-European online platform hosting decision support systems and associated resources for integrated pest management," can be adjusted to better reflect the paper's primary focus on technical implementation, architecture, and process. When I first read the title, I was expecting to read about a catalog of DSS associated with IPM.
2. Figure 1 is complicated and is only referenced once in the text. Text only explains the central server portion of the figure. Either need more explanation in text or in figure caption.
3. Figure 2 seems unnecessary and is a bit complicated for not really describing a complicated concept. You can probably explain in text that there are a variety of levels that DSS can interact with an IPM Decision Platform as you have examples with the Case Studies.
4. The article is structured as a report on the development of the platform, the APIs it uses, and the process for integrating a new DSS, rather than a deep dive into the content of the DSS portfolio. However, there would still be value in including a table that summarized the 79 DSSs currently integrated or partially integrated into the platform. A summary of these DSSs can provide a baseline of what is currently available (crops, pests, countries). Also, you mention a few times that DSSs need to be provided by “reputable sources” (e.g., pages 9, 10) but “reputable” is hard to define. If you summarized the contributors of the 79 current DSSs, perhaps you can start framing in who is considered “reputable” – as these folks have already passed that criteria.
5. The article is very term heavy. I found myself trying to learn a new set of terms and trying to figure out how they all work together. In some cases, terms were introduced but never really explained how they fit into the larger picture. Here is a list I tagged as reading it (I may have missed some, including several terms in Figure 1 that do not show up in the text). Can a table be added that has a definition of these terms?

- Crop Protection Online (CPO, 2025): A commercial service in Denmark, managed by Aarhus University and SEGES, which hosts a series of Decision Support Systems.
- DSS Adaption Dashboard (page 9): ??
- DSS USE Dashboard (page 9): ??
- It is not entirely clear the differences (if there are any) between DSS, IPM DSS, and IPM Decision Platform.  The name of the IPM DSS is the IPM Decision Platform and it might be best to stick with the name through the paper.
- GitHub: Where a repository of the IPM Decisions Platform's central source code is available.
- Horizon 2020 research and innovation programme: The European Union programme that provided funding for the IPM Decisions project.
- IPM Adviser: A free web-based tool that implements the IPM DSS typology to provide standardized, comparable information on available IPM DSS.
- IPM Decisions Platform (or IPM Decisions Platform, 2025): The main subject of the article, described as a Pan-European online platform and the first open-access pan-European 'click and go' marketplace for Integrated Pest Management Decision Support Systems (IPM DSS). 
- IPM Decisions Factory (page 9): ??
- IPMWORKS (2020) project: A sister project to IPM Decisions that established an IPM demonstration farm network.
- Open-Meteo.com's APIs (2025): A service used by the IPM Decisions Platform's Weather API to provide hourly weather data.
- VIPS (VIPS, 2025): A platform that has been running in Norway for over 20 years, forecasting pest risk levels.
  6. Strengthen Conclusion: To strengthen the paper and address the reader's inevitable question about real-world impact—"Does it help?"—the Conclusion section should acknowledge the boundary between the paper's scope and the project's ultimate goal. You should bridge the gap between the platform's technical success and its intended effect on user behavior and sustainability. The paper, as an architectural report, focuses on the "supply side" (making the tool available), but the Conclusion needs to make a forward-looking statement that acknowledges the missing "demand side" evidence. In the final paragraph of the Conclusion, the authors could add a statement that a) Acknowledge the Gap: Explicitly state that the paper confirms the platform's technical feasibility but does not yet measure its efficacy in changing farming practices; b) Define the Next Critical Step: Clearly articulate that future work is essential to assess real-world user uptake (e.g., how many farmers/consultants are using it); and c) Specify the Impact Metric: Conclude by highlighting the necessity of quantifying the economic and environmental payoff—specifically, by measuring the platform's impact on IPM adoption rates and pesticide usage reduction (and potentially referencing relevant impact studies, like Helps et al. 2024, to set a benchmark).
  7. Consulting section what covered the user experience links to terms and conditions, but it should include a discussion of the storage and use of user data.  I was surprised not to see the sharing of personal farm data as a barrier to uptake, but maybe there is a different culture around this in the EU – which would also be helpful to discuss.
  8. Use of capitalization is inconsistent. Please fix. For example, you have both IPM Decisions **P**latform and IPM Decisions **p**latform. You also have both **P**latform and **p**latform.  

 

1. Several grammatical errors.
  1. Plain Language Summary has several misspelled words including…
    1. dieases
    2. unnecassary
    3. treatements (twice)
    4. intergrated
    5. recomendations
    6. devlopment
    7. deomonstraiotns
    8. … **update** of IPM DSS in Europe… should be … **uptake** of DSS in Europe…
  2. Page 4 - Decision Support Systems for Integrated Pest Management
    1. …important tool in supporting pesticide **s** use… (delete s in pesticides)
  3. Page 5 - Platform design and development
    1. Capitalize the API in “Application Programming Interfaces” the first time you mention it (bottom of first column)
    2. Replace “Application Programming Interfaces (APIs) with “APIs” at bottom of second column
  4. Page 6
    1. Change “data is” to “data are” in first sentence of second paragraph
    2. Change “If a special weather parameter like for instance leaf wetness”… to “If a special weather parameter, for instance leaf wetness,…
    3. Delete “streams” in last sentence in first column
  5. Figure 2
    1. Spacing in box saying “Do you want to use the platform interface?”
  6. Page 8
    1. Change “Work has subsequently been completed to extend access to these models to users in **Belgium. Through **provision of additional historic soil temperature data.” To “Work has subsequently been completed to extend access to these models to users in **Belgium, through **provision of additional historic soil temperature data.”
  7. Page 9
    1. Change “…IPM Decisions platform (currently available for Metos, MeteoBot, and Fruitweb) **this** data can also be used.” to “…IPM Decisions platform (currently available for Metos, MeteoBot, and Fruitweb) **these** data can also be used.”
    2. Change  “…This may include adding details on crop drilling date, growth stages, plant protection product (PPP)… to “This may include adding details on crop drilling date, growth stages, pesticide…”
    3. Delete “made” in the sentence “Users registering an account on the IPM Decisions platform are required to accept the platform Terms and Conditions (IPM Decisions, 2023c), and are made alerted that the platform makes DSS available from external DSS developers.”
  8. Page 10
    1. Change “Therefore, it is not a requirement that all these criteria are met for a DSS to be available through this Platform.” to “Therefore, not all these criteria are required for a DSS to be available through this Platform.”
    2. Change “The DSS can be used to assist (not replace) experienced crop managers in decision making process,…” to “The DSS can be used to assist (not replace) experienced crop managers in thedecision making process,…”
    3. Question: You state - Farmer groups and research organisations are encouraged to test the DSS in the field, amend the DSS to suit local conditions if necessary, and share their experiences. **How can they share their experiences? **
    4. Question: You state … several sites and seasons, including field trials testing DSS for septoria, grape downy mildew and apple scab. **Should more detail be included in the septoria testing. Full name? Crop?**
    5. Change “By providing a framework to estimate the economic value of DSS in different regions where pest conditions may be different, users can be provided with greater knowledge about the potential benefits and risks of any given DSS. While there may be many DSS available for a particular pest/cropping system, most of them are tested and recommended only in the country…” to “By providing a framework to estimate the economic value of DSS in different regions where pest conditions may be different, users can be provided with greater knowledge about the potential benefits and risks of any given DSS. While there may be many DSS may be available for a particular pest/cropping system, most of them are tested and recommended only in the country…”
    6. Change “The resulting differences in yield, cost of production and treatment frequency index (TFI) were used to evaluate the overall value of implementing DSS.” to “The resulting differences in yield,production cost of production and treatment frequency index (TFI) were used to evaluate the overall value of implementing DSS.”
    7. Change “Data was collected on total weight of pesticide active substance used at the national/European level…” to “Data were collected on total weight of pesticide active substance used at the national/European level…”
    8. Delete “their” in this sentence – “The use of DSS provides growers with the opportunity to reduce theirpesticide usage,…”
    9. Change “of” to “in the” in this sentence - An increase of in the application of IPM DSS by 25–50% in wheat, potatoes and grapes…”
  9. Page 11
    1. Change “The IPM DSS strategy was compared with several other growth stage-based treatment applications, and data was collected to determine the effectiveness of each approach in managing priority wheat diseases.” to “The IPM DSS strategy was compared with several other growth stage-based treatment applications, and data were collected to determine the effectiveness of each approach in managing priority wheat diseases.”
2.
  1.
    1. Change “In each of seven trials, two disease management…” to “In each of seven trials, two disease management…”
    2. Change “Combining the consultation of a decision support system with a farmer or advisors knowledge of disease epidemics shows great potential in optimising fungicide programmes.” to “Combining the consultation of a DSS with a farmer or advisor’s knowledge of disease epidemics shows great potential in optimising fungicide programmes.”
  2. Page 12
    1. Question: Your sentence saying “In some regions, long term investment in the development of IPM DSS has generated a range of systems and associated public and/or or commercial platforms, while elsewhere access is highly restricted and limited to commercial enterprises.” **Does “commercial” mean “pay to play” or does “commercial” indicate that these systems are from the private sector/industry?**
  3. Page 14
    1. Change in the Data Availability section – “No data is associated with this article. When data was collected as part of individual studies and survey, data is detailed in associated publications.” to “No data are associated with this article. When data were collected as part of individual studies and survey, data are detailed in associated publications.”

Where applicable, are recommendations and next steps explained clearly for others to follow? (Please consider whether others in the research community would be able to implement guidelines or recommendations and/or constructively engage in the debate)

Yes

Does the article adequately reference differing views and opinions?

Yes

Are all factual statements correct, and are statements and arguments made adequately supported by citations?

Yes

Is the rationale for the Open Letter provided in sufficient detail? (Please consider whether existing challenges in the field are outlined clearly and whether the purpose of the letter is explained)

Yes

Is the Open Letter written in accessible language? (Please consider whether all subject-specific terms, concepts and abbreviations are explained)

Yes

Reviewer Expertise:

Plant Pathologist, IPM Coordinator, Co-Director of North Central IPM Center and Crop Protection Network

We confirm that we have read this submission and believe that we have an appropriate level of expertise to confirm that it is of an acceptable scientific standard.
